# Serological surveillance of *Trypanosoma evansi* in Kazakhstani camels by complement fixation and formalin gel tests

**DOI:** 10.3389/fvets.2025.1661387

**Published:** 2025-09-23

**Authors:** Zhandos Abay, Zhadra Kudaibergenova, Alim Bizhanov, Maksat Serikov, Salika Berdiakhmetkyzy, Altynai Arysbekova, Roza Aitlessova, Temir Smadil, Serikbay Kadyrov, Berik Lessov, Rano Sattarova, Serik Kanatbayev, Bolat Shalabayev, Kuandyk Shynybayev, Nurkuisa Rametov, Nurlan Akhmetsadykov, Han Sang Yoo, Nurgul Sikhayeva, Aralbek Rsaliyev, Yergali Abduraimov, Markhabat Kassenov, Ainur Nurpeisova

**Affiliations:** ^1^Kazakh Scientific Research Veterinary Institute LLP, Almaty, Kazakhstan; ^2^Kazakh National Agrarian Research University, Almaty, Kazakhstan; ^3^Department of Geospatial Engineering, Satpaуev Kazakh National Research Technical University, Almaty, Kazakhstan; ^4^Antigen LLP, Almaty, Kazakhstan; ^5^College of Veterinary Medicine, Seoul National University, Seoul, Republic of Korea; ^6^Qazbiopharm JSC, Astana, Kazakhstan

**Keywords:** *Trypanosoma evansi*, surra, camel, seroprevalence, complement fixation test, formalin gel test, vector-borne diseases, antibodies

## Abstract

**Introduction:**

Surra, caused by *Trypanosoma evansi* (*T. evansi*), is a significant vector-borne disease of camels that leads to substantial economic losses in affected regions. This study was conducted to determine the seroprevalence of surra among dromedary (*Camelus dromedarius*) and Bactrian (*Camelus bactrianus*) camels in Kazakhstan.

**Methods:**

A cross-sectional survey was carried out between January and May 2024 in the Mangystau, Kyzylorda, and Turkestan regions. A total of 2,773 camel serum samples (1,045 males and 1,728 females) were collected and tested using the complement fixation test (CFT) and the formol gel test (FGT). Chi-square tests were applied to assess differences across age groups, sexes, and regions.

**Results:**

Antibodies against *T. evansi* were detected in 113 camels (4.07%; 95% CI: 3.36–4.86) by CFT and in 276 camels (9.95%; 95% CI: 8.88–11.13) by FGT. Seroprevalence increased with age, with the highest rates observed in camels older than 12 years (5.93% by CFT and 26.27% by FGT). Females had significantly higher prevalence than males (CFT: 4.69% vs. 3.06%; FGT: 10.47% vs. 9.09%, *p* = 0.046). Regional variation was also noted, with the highest prevalence detected in Mangystau by FGT (65.0%).

**Discussion:**

These findings confirm that camel surra is endemic in the surveyed regions of Kazakhstan. Both serological tests proved useful for large-scale screening of *T. evansi*, and the FGT, due to its higher sensitivity, is recommended as the preferred tool for field surveillance.

## 1 Introduction

Camel husbandry holds significant economic, social, and cultural importance in Kazakhstan, particularly within pastoral communities. Camels provide essential resources, including meat, milk, wool, and transportation, contributing considerably to rural livelihoods and agricultural stability. However, the health and welfare of camels are threatened by a wide range of pathogens, including bacterial, viral, and parasitic agents, which negatively impact productivity and survival. Among these, parasitic infections play a major role, with trypanosomoses recognized as particularly important due to their chronic nature, economic impact, and potential zoonotic relevance (1–3).

*Trypanosoma evansi (T. evansi),* the causative agent of surra, is a chronic vector-borne parasite that affects various domestic animals, including horses, donkeys, dogs, camels, sheep, and goats, and is also a potential human pathogen, resulting in anemia, progressive weight loss, reproductive failure, reduced productivity, and, in severe cases, mortality (2, 4–7). Surra is widely distributed globally, predominantly across regions of Asia, North Africa, Central, and South America, where environmental conditions favor vector survival and parasite transmission (1, 2).

Despite the large camel population and documented outbreaks of trypanosomiasis in Kazakhstan, the disease is poorly documented and there are few literature sources. To date, no peer-reviewed data exist on camel trypanosomiasis in Kazakhstan, and the only relevant publication is the study by Claes et al. (4), which investigated equine trypanosomosis. This highlights a substantial knowledge gap and underscores the importance of targeted research on *T. evansi* in camels in Kazakhstan.

Diagnosis of trypanosomiasis can be clinical, parasitological, serological, or molecular (8–10). In most cases, a single diagnostic method does not unequivocally identify the different statuses of infection. These include “non-infected,” “asymptomatic carrier,” “sick infected,” “cured/not cured,” and/or “multi-infected.” Therefore, integrative approaches combining parasite detection, molecular methods, and antibody detection, along with epizootiological information, are needed (11, 12).

Control of surra still relies mainly on the observation of clinical signs and subsequent treatment of sick animals, which is inefficient and results in high morbidity and mortality of undiagnosed animals that in the meantime act as reservoirs. Indirect diagnosis is possible through detection of specific antibodies in the mammalian hosts. Currently available antibody detection tests include the immune trypanolysis assay (13), an enzyme-linked immunosorbent assay for *T. evansi* (ELISA/*T. evansi*) (14), card agglutination test for trypanosomiasis (CATT/*T. evansi*) (1), latex agglutination test, and simpler methods for field mass screening such as the complement fixation test (CFT) and the formol gel test (FGT). Definitive diagnosis of *T. evansi* infection is achieved by microscopic demonstration of the parasite, which however suffers from low sensitivity due to low parasitemia. The choice of one or several tests is guided by epidemiological requirements, technical feasibility, and cost-effectiveness (15, 16).

Serological tests such as CFT and FGT, despite their limitations in sensitivity and specificity, have been widely used for large-scale surveillance in camels because they are simple, inexpensive, and applicable under field conditions.

Our current study aimed to investigate the seroprevalence of *T. evansi* infection in camels in Kazakhstan using CFT and FGT. The result of this study will be useful for the surveillance and control of *T. evansi* infection.

## 2 Materials and methods

### 2.1 Study area and sampling

**Figure 1:**
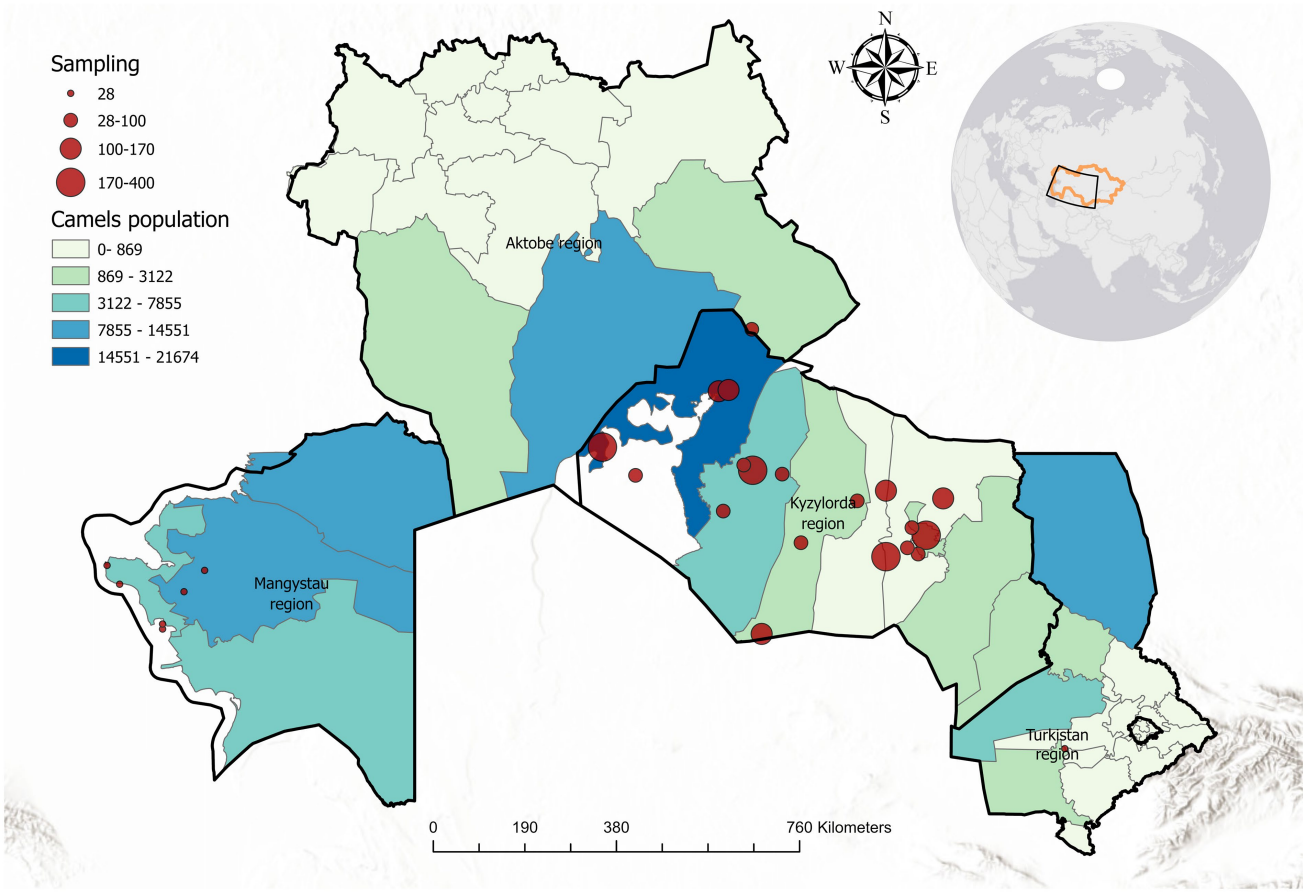
Map of the study area in Kazakhstan, highlighting Mangystau, Kyzylorda, and Turkestan regions. Sampling locations are marked with red circles (circle size proportional to number of samples at that location). Camel population density is indicated by a color gradient from light green (low density) to dark blue (high density).

A cross-sectional serological survey was carried out in three regions of southwestern Kazakhstan (Mangystau, Kyzylorda, and Turkestan), which are prominent camel-breeding areas. The climate of these regions is extremely continental and arid, with hot, dry summers (often exceeding 40–45 °C) and cold winters, with only mild spells. Annual precipitation is very low (100–150 mm), and the landscape is dominated by desert vegetation. Figure 1 shows the geographic distribution of the sampling locations across the three regions.

Sampling was conducted from January to May 2024 in collaboration with local veterinary services. Within each region, several districts with large camel populations were identified, and herds were selected to maximize geographical coverage. In each selected herd, all available camels that met the inclusion criteria were sampled rather than only a subset, thereby avoiding clustering bias. When herds were very large (>200 animals), at least 30–40% of the animals were sampled proportionally across age and sex categories to ensure representativeness.

In total, 2,773 camels were examined, comprising 100 Bactrian camels from Mangystau and 2,673 dromedary camels from Kyzylorda and Turkestan. The sampled population included 1,045 males and 1,728 females, with ages ranging from under 3 years to over 12 years. Only camels older than 1 year and with no anti-trypanosomal treatment in the prior 6 months were included in the study.

### 2.2 Sample size determination

The minimal sample size required for this study was determined according to the random sampling method described by Thrusfield (17, 18). Because no previous estimates of *T. evansi* prevalence in camels were available for Kazakhstan, we applied the conservative assumption of an expected prevalence (P) of 50%, which maximizes the product P(1–P), with 5% precision at the 95% confidence level. The formula used for sample size calculation was given in Equation (1):


(1) $$N=\frac{{\left(1.96\right)}^{2}*P(1-P)}{d^{2}}$$

where *N* is the required sample size, *P* is the expected prevalence, and *d* is the desired absolute precision.

This calculation indicated a minimum requirement of 384 animals. In practice, we collected a total of 2,773 samples, which considerably exceeded this threshold and ensured high precision of prevalence estimates. The narrow 95% confidence intervals around the overall prevalence values (CFT 4.07, 95% CI: 3.36–4.86; FGT 9.95, 95% CI: 8.88–11.13) confirm the adequacy of the realized sample size.

### 2.3 Serological testing

Blood samples were collected from the jugular vein into vacutainer tubes and allowed to clot at ambient temperature. Sera were separated by centrifugation and stored at −20 °C until testing. All samples were tested for *T. evansi*-specific antibodies using two serological assays: the CFT and the FGT. Both tests were carried out according to standard protocols commonly used for surra surveillance in the region.

#### 2.3.1 Complement fixation test

For the CFT, a commercial *T. evansi* antigen kit (KazSRVI, Kazakhstan) was used. The quantitative protein concentration of the antigen was standardized at 1 mg per cm^3^. Guinea pig serum was used as the source of complement (batch No. 15, expiry date December 2025).

Test serum were incubated with antigen and complement at 37 °C for 20 min, followed by the addition of sensitized sheep red blood cells. Results were expressed based on the degree of haemolysis inhibition (HI) at a 1/5 serum dilution, using the following scale: 0, trace, 1+, 2+, 3+, or 4+, corresponding to 0%, trace, 25, 50, 75%, or 100% of unlysed red cells, respectively. Qualitative interpretation (positive or negative) was determined at the 1/5 dilution, with ≥2 + considered positive.

Positive and negative control serum were included in each run to calibrate the assay and ensure quality control. Positive controls contained active complement, whereas negative controls consisted of inactivated serum.

#### 2.3.2 Formol gel test

The FGT was performed by adding 0.1 cm^3^ of a 40% formalin solution (aqueous solution of formaldehyde, 40% v/v, equivalent to 37% w/w) to 1.0 cm^3^ of camel serum, giving a serum-to-formalin ratio of 10:1. The tubes were gently mixed and left at room temperature for observation. Reactions were read after 48 h.

The test outcome was interpreted qualitatively as the presence or absence of gel formation or turbidity. To minimize observer bias, all tubes were coded, and results were assessed by a single blinded observer who was unaware of the identity (positive or negative) of the samples. After readings were completed, the tubes were decoded, and diagnostic performance indicators were calculated, including sensitivity (the proportion of truly infected animals testing positive), specificity (the proportion of non-infected animals testing negative), positive predictive value, and negative predictive value. Positive and negative control serum were included in each test batch to ensure reliability of results.

### 2.4 Data analysis

Seroprevalence was calculated as the proportion of positive samples with corresponding 95% confidence intervals (CI). Camels were stratified by age group (juvenile <3 years, young adult 3–6 years, mature adult 6–12 years, and old >12 years), sex, and geographic region for comparative analysis. Pearson’s chi-square tests were applied to assess differences in seroprevalence across categories, with *p* < 0.05 considered statistically significant. To evaluate the agreement between CFT and FGT, cross-tabulation was performed and Cohen’s kappa statistic (*κ*) was calculated, with values interpreted as poor (<0.20), fair (0.21–0.40), moderate (0.41–0.60), good (0.61–0.80), and almost perfect (>0.80) agreement. Observed and expected agreements were also reported. All statistical analyses were performed using SPSS 26.0. A map of sampling locations was produced using ArcGIS Pro 3.4 to visualize the spatial coverage of the study (Figure 1).

### 2.5 Ethical considerations

The study was approved by the Local Biological Ethics Committee of the Kazakh Research Veterinary Institute (Protocol #1, 14 July 2023). Informed consent was obtained from all camel owners and participating veterinarians prior to blood sample collection.

## 3 Results

**Figure 2:**
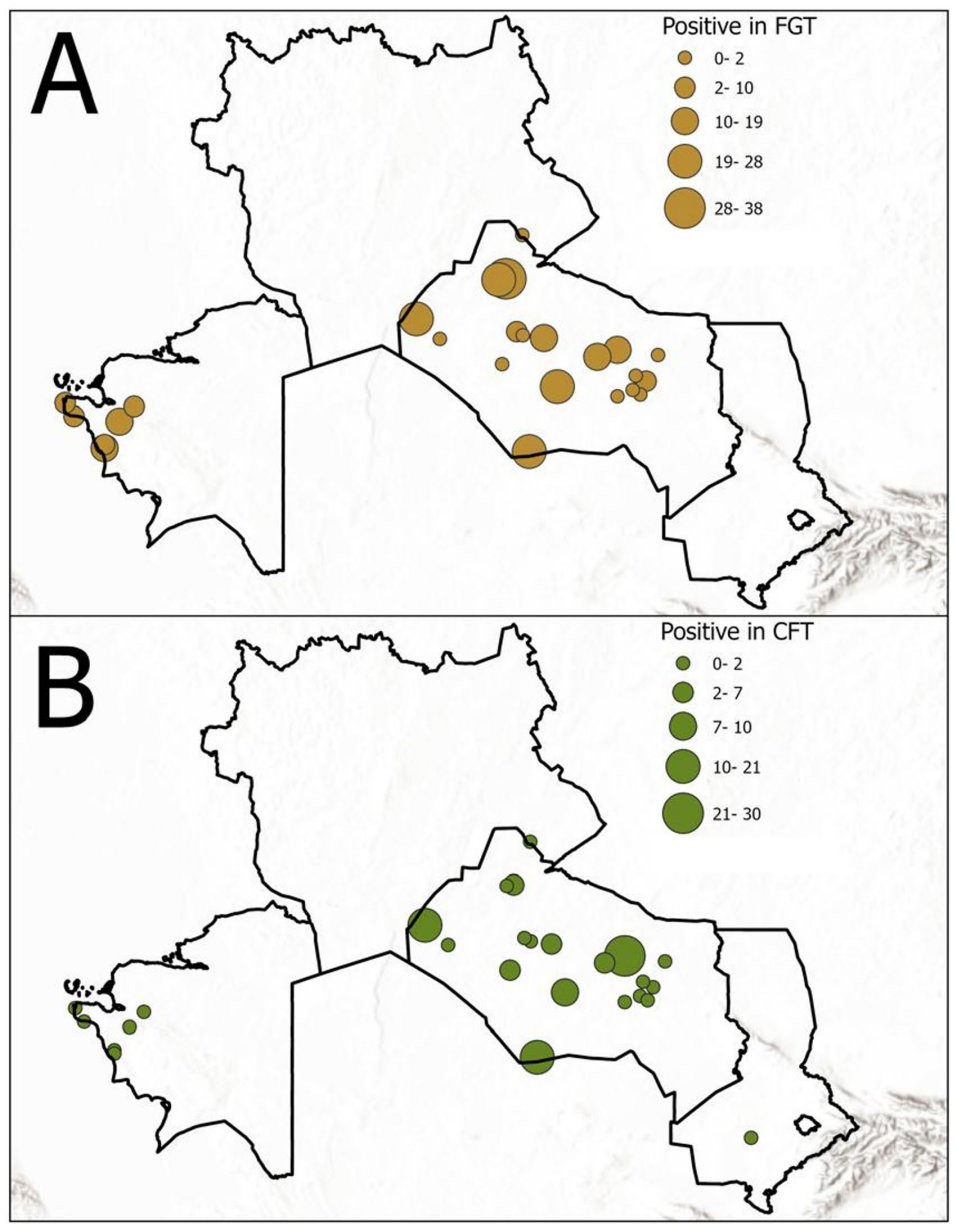
Seroprevalence of *T. evansi* in the surveyed camels by serological test: **(A)** FGT results and **(B)** CFT results. The chart illustrates the higher detection rate of FGT compared to CFT. FGT, Formol gel test; CFT, Complement fixation test.

Out of 2,773 camel sera tested, 113 (4.07%) were positive for *T. evansi* antibodies by CFT, and 276 (9.95%) were positive by FGT (Figure 2).

Thus, the FGT detected more than twice as many seropositive camels as the CFT. The seroprevalence results stratified by age group and sex are summarized in Tables 1, 2.

**Table 1:** Seroprevalence of *T. evansi* by age group (Kazakhstan, 2024).

Age group	Number tested (*N*)	CFT-positive		FGT-positive	
		*n*, (%)	95% CI	*n*, (%)	95% CI
Juveniles (<3 years)	813	22 (2.70)	1.79–4.06	77 (9.47)	7.64–11.68
Young adults (3–6 years)	1,057	53 (5.02)	3.71–6.77	85 (8.04)	6.35–10.14
Mature adults (6–12 years)	785	31 (3.95)	3.09–5.10	83 (10.57)	9.08–12.24
Old camels (>12 years)	118	7 (5.93)	2.75–12.36	31 (26.27)	18.22–35.05
Total	2,773	113 (4.07)		276 (9.95)	

FGT, Formol gel test; CFT, Complement fixation test.

**Table 2:** Seroprevalence of *T. evansi* by sex.

Sex	Number tested (*N*)	CFT-positive		FGT-positive	
		*n*, (%)	95% CI	*n*, (%)	95% CI
Male	1,045	32 (3.06)	2.18–4.29	95 (9.09)	7.49–10.99
Female	1,728	81 (4.69)	3.79–5.79	181 (10.47)	9.12–12.01
Total	2,773	113 (4.07)		276 (9.95)	

FGT, Formol gel test; CFT, Complement fixation test.

Age-related differences in seroprevalence were statistically significant (*χ*^2^, *p* < 0.0001). Old camels (>12 years) showed the highest proportional prevalence, with 5.93% (95% CI: 2.75–12.32) positive by CFT and 26.27% (95% CI: 18.49–35.78) positive by FGT. Mature adults (6–12 years) recorded 3.95% (95% CI: 3.06–5.09) by CFT and 10.57% (95% CI: 9.08–12.28) by FGT, while young adults (3–6 years) showed 5.02% (95% CI: 3.69–6.79) and 8.04% (95% CI: 6.35–10.13), respectively. Juveniles (<3 years) exhibited the lowest prevalence, with 2.70% (95% CI: 1.78–4.08) in CFT and 9.47% (95% CI: 7.64–11.68) in FGT (Table 1). Despite the smaller sample size in the oldest age group, the high rates observed confirm that older camels are more likely to have been exposed to *T. evansi*.

Sex-related differences were also observed (Table 2). Seroprevalence was significantly higher in females compared to males, both in CFT (4.69%; 95% CI: 3.78–5.80 vs. 3.06%; 95% CI: 2.17–4.30) and in FGT (10.47%; 95% CI: 9.10–12.03 vs. 9.09%; 95% CI: 7.48–11.00). This sex-related difference in infection rate was statistically significant (*p* = 0.046). In absolute numbers, 81 females versus 32 males were CFT-positive, and 181 females versus 95 males were FGT-positive. The higher infection rate in females could be associated with factors such as greater exposure to vectors (for example, around watering or feeding sites), physiological stress from reproduction, or sex-based differences in immune response. Overall, the FGT identified substantially more positive cases than the CFT across all age groups and in both sexes. In every category, some camels that tested negative by CFT were positive by FGT. This discrepancy suggests that the FGT has higher analytical sensitivity, potentially detecting low-level or older infections that the CFT might miss. However, it also raises the possibility that FGT-positive/CFT-negative results could include animals with residual antibodies from earlier infections or non-specific reactions. No camels were found CFT-positive while FGT-negative in this study.

Seroprevalence varied significantly across the three surveyed regions (Table 3). In Mangystau, the proportion of seropositive camels was 5.0% (95% CI: 2.15–11.18) by CFT and 65.0% (95% CI: 55.13–73.76) by FGT. In Kyzylorda, prevalence was 4.01% (95% CI: 3.33–4.82) by CFT and 7.83% (95% CI: 6.86–8.91) by FGT, while in Turkestan prevalence reached 7.14% (95% CI: 1.98–22.65) by CFT and 14.29% (95% CI: 5.70–31.49) by FGT. These findings indicate marked regional heterogeneity in the distribution of surra in Kazakhstan.

**Table 3:** Seroprevalence of *T. evansi* in camels by geographic region.

Region	Number tested (*N*)	CFT-positive		FGT-positive	
		*n*, (%)	95% CI	*n*, (%)	95% CI
Mangystau	100	5 (5.0%)	2.15–11.18	65 (65.0%)	55.25–73.64
Kyzylorda	2,645	106 (4.0%)	3.32–4.82	207 (7.8%)	6.86–8.91
Turkestan	28	2 (7.1%)	1.98–22.65	4 (14.3%)	5.70–31.49
Total	2,773	113 (4.07%)		276 (9.95%)	

FGT, Formol gel test; CFT, Complement fixation test. A district-level breakdown of prevalence by area is provided in Supplementary Table S1.

A more detailed district-level breakdown is provided in Supplementary Table S1. In Kyzylorda, the highest prevalence by FGT was observed in Karmakshy (19.06%; 95% CI: 15.14–23.72) and Aral (13.43%; 95% CI: 11.06–16.24) districts, while very low levels were recorded in Syrdarya (2.64%; 95% CI: 1.29–5.35) and Kyzylorda (0.55%; 95% CI: 0.19–1.59) districts. In Mangystau, extremely high prevalence was detected in Munaily (90.0%; 95% CI: 74.38–96.54) and Mangystau (72.5%; 95% CI: 57.19–83.89) districts. In Turkestan, Shardara district showed moderate prevalence (14.3%; 95% CI: 5.70–31.49), although the sample size was small (*n* = 28). These district-level findings underscore strong local variation, which may reflect ecological conditions, herd management practices, and vector abundance.

Agreement between the two serological assays was further evaluated (Table 4). Overall, 92.4% of samples showed concordant results between CFT and FGT. The Cohen’s kappa statistic was 0.41, indicating moderate agreement. As expected, FGT detected additional positive samples that were negative in CFT, reflecting its higher sensitivity but lower specificity.

**Table 4:** Agreement between CFT and FGT for detection of *T. evansi* antibodies in camels.

CFT / FGT	FGT positive	FGT negative	Total
CFT positive	90	23	113
CFT negative	186	2,474	2,660
Total	276	2,497	2,773

## 4 Discussion

This study provides the first seroepidemiological evidence of *T. evansi* infection in camels from Kazakhstan, filling a major knowledge gap in Central Asia. The overall prevalence detected in our survey (4.07% by CFT; 9.95% by FGT) indicates that surra is present at endemic levels, although with strong local variation. These results are broadly comparable with reports from ecologically similar regions such as Iran (7.5–12.3%) (2) and Algeria (8.7%) (16, 18), but considerably lower than in some African countries where prevalence may exceed 20–30% (1). Such comparisons highlight that the epidemiology of surra is strongly influenced by ecological conditions, management practices, and vector abundance.

As expected, the FGT identified more positive samples than the CFT across all categories. This finding is consistent with earlier reports that the FGT, although less specific, is more sensitive in detecting residual or cross-reacting antibodies (9). The CFT is considered more specific but may miss chronic or low-level infections due to waning antibody titers. Importantly, the possibility of cross-reactivity with other trypanosome species and lingering antibodies post-treatment cannot be excluded. In endemic areas, animals may retain detectable antibodies for months or years, which inflates seroprevalence without necessarily indicating active infection. This underlines the need for cautious interpretation of serological results and the complementary use of molecular tests in future studies.

The agreement analysis between the two serological tests indicated an overall concordance of 92.4% but only moderate agreement (*κ* = 0.41). This finding reflects the different diagnostic properties of the assays: while CFT is more specific, FGT identifies a higher number of positive cases due to its greater sensitivity. Similar patterns have been reported in other endemic regions, where FGT tends to overestimate prevalence compared to CFT or ELISA. These results emphasize the importance of carefully selecting the appropriate serological tool depending on whether the priority is screening (sensitivity) or confirmation (specificity).

Age-related differences were pronounced. Old camels (>12 years) showed the highest proportional prevalence (26.3% by FGT), followed by mature adults, young adults, and juveniles. This pattern reflects cumulative exposure to *T. evansi* over time, as older animals are more likely to have been repeatedly exposed to vectors. Similar age-related increases have been reported in Algeria and Ethiopia (19). In contrast, juveniles consistently showed the lowest prevalence, likely reflecting shorter exposure duration and possibly stronger veterinary supervision in younger animals.

Seroprevalence was also higher in females compared to males, a pattern observed in previous studies (20, 21). Possible explanations include physiological stress during pregnancy and lactation, as well as husbandry practices where females are retained longer in herds for breeding and milk production, thus accumulating greater lifetime exposure.

Marked regional heterogeneity was evident, with extremely high prevalence in Mangystau (65% by FGT), moderate levels in Turkestan (14.3%), and relatively low prevalence in Kyzylorda (7.8%). The district-level breakdown (Supplementary Table S1) further highlights this variation. In Kyzylorda, prevalence ranged from as low as 0.55% in Kyzylorda district to as high as 19.1% in Karmakshy. Similarly, in Mangystau, prevalence exceeded 70% in Mangystau and Munaily districts but was only 30% in Tupkaragan. These findings illustrate how local ecological conditions – such as vector density near rivers and pastures – may strongly shape infection risk. Comparable intra-regional variation has been reported in Iran and Pakistan (22).

Transmission of *T. evansi* in camels is primarily mechanical, mediated by biting flies such as Tabanus spp., Stomoxys spp., and Haematobia spp. Vector ecology is strongly linked to temperature, rainfall, and vegetation cover, which create suitable habitats for fly breeding and activity (2). Our survey was conducted between January and May, prior to the peak summer months when tabanids are most active. Therefore, some seasonal bias cannot be ruled out, as prevalence may differ if sampling is performed later in the year during maximum vector activity. Longitudinal studies across different seasons are needed to capture this dynamic.

Globally, a wide range of diagnostic tools are used for surra surveillance, including ELISA, the card agglutination test (CATT/*T. evansi*), latex agglutination, and molecular assays such as PCR (2). While serological tests such as CFT and FGT remain practical for large-scale surveys, they cannot distinguish between past and current infections and may be influenced by cross-reactions. The incorporation of molecular tools in future studies would allow confirmation of active infection and a more precise understanding of disease dynamics.

An additional challenge in interpreting serological data is the frequent use of trypanocidal drugs by camel owners. Indeed, the majority of camels in the surveyed farms had a history of treatment with locally produced urea-derivative drugs or with Deminkel (Belgium). Such practices may reduce parasitemia and clinical signs, but antibodies often remain detectable for extended periods. This complicates the distinction between current and past infections. Similar observations were reported by Boushaki D. et al. (23), who emphasized that prior chemotherapeutic intervention can bias prevalence estimates by lowering parasite detection while maintaining serological positivity.

The main limitations of our study include the relatively small sample size in some districts (e.g., Shardara, *n* = 28), the exclusive reliance on serological tests, and the lack of longitudinal data across different seasons. While molecular methods such as PCR and sequencing were beyond the scope and objectives of this survey, we acknowledge this as a limitation and emphasize that future studies should incorporate molecular confirmation to complement serological data and provide a more comprehensive picture of strain diversity and infection dynamics. Despite these limitations, our findings establish baseline data for surra in Kazakhstan and demonstrate clear patterns of age-, sex-, and region-related risk. These results emphasize the importance of tailored surveillance strategies and the need to integrate serological and molecular diagnostics, as well as vector monitoring, in order to design effective control measures for surra in camels.

In summary, this study provides important surveillance data on *T. evansi* in Kazakhstan’s camel population and evaluates the practical performance of two serological tests in the field. The notable differences between CFT and FGT outcomes highlight that a one-test strategy may underdetect surra. Field programs might achieve better sensitivity by using the FGT for initial screening, but they should be aware of its limitations and consider confirmatory testing. Our findings also indicate that certain groups (especially adult female camels) have higher infection rates, which could inform targeted interventions.

## 5 Conclusion

The surveillance conducted in three regions of Kazakhstan revealed a significant prevalence of *T. evansi* infection in camels, detected through serological testing. The CFT and FGT proved to be useful tools for large-scale seromonitoring of surra in camels, with the FGT demonstrating a higher overall sensitivity in this setting. These results underline the importance of using both tests in complement: the FGT for broad surveillance and the CFT (or another specific assay) for confirming active cases. Strengthening surra control in Kazakhstan will require continued monitoring of camel herds using reliable diagnostic methods, combined with the development of improved diagnostic assays for greater accuracy. Further, implementing vector control and other preventive measures will be essential to mitigate the impact of surra on camel health and the livelihoods of pastoral communities.

## Data Availability

The original contributions presented in the study are included in the article/Supplementary material, further inquiries can be directed to the corresponding author.

## References

[1] Aregawi WG Agga GE Abdi RD Büscher P. Systematic review and meta-analysis on the global distribution, host range, and prevalence of *Trypanosoma evansi*. Parasit Vectors. (2019) 12:1–25. doi: 10.1186/s13071-019-3311-4, PMID: 30704516 PMC6357473

[2] Desquesnes M Holzmuller P Lai D-H Dargantes A Lun Z-R Jittaplapong S. *Trypanosoma evansi* and Surra: a review and perspectives on origin, history, distribution, taxonomy, morphology, hosts, and pathogenic effects. Biomed Res Int. (2013) 2013:194176. doi: 10.1155/2013/194176 24024184 PMC3760267

[3] Abdel-Hakeem SS Megahed G Al-Hakami AM Tolba ME Karar YF. Impact of trypanosomiasis on male camel infertility. Front Vet Sci. (2025) 11:1506532. doi: 10.3389/fvets.2024.1506532, PMID: 39885842 PMC11780594

[4] Claes F Ilgekbayeva GD Verloo D Saidouldin TS Geerts S Buscher P. Comparison of serological tests for equine trypanosomosis in naturally infected horses from Kazakhstan. Vet Parasitol. (2005) 131:221–5. doi: 10.1016/j.vetpar.2005.05.001, PMID: 15951112

[5] Alanazi AD Puschendorf R Salim B Alyousif MS Alanazi IO Al-Shehri HR. Molecular detection of equine trypanosomiasis in the Riyadh Province of Saudi Arabia. J Vet Diagn Invest. (2018) 30:942–5. doi: 10.1177/1040638718798688, PMID: 30204053 PMC6505846

[6] Al Malki JS Hussien NA. Molecular characterization of Trypanosoma evansi, T. Vivax and T. Congolense in camels (*Camelus dromedarius*) of KSA. BMC Vet Res. (2022) 18:45. doi: 10.1186/s12917-022-03148-0, PMID: 35042521 PMC8764778

[7] Mossaad E Salim B Suganuma K Musinguzi P Hassan MA Elamin EA. *Trypanosoma vivax* is the second leading cause of camel trypanosomosis in Sudan after *Trypanosoma evansi*. Parasit Vectors. (2017) 10:1–10. doi: 10.1186/s13071-017-2117-5, PMID: 28403897 PMC5390396

[8] Kivali V Kiyong’a AN Fyfe J Toye P Fèvre EM Cook EA. Spatial distribution of trypanosomes in cattle from Western Kenya. Front Vet Sci. (2020) 7:554. doi: 10.3389/fvets.2020.00554 33005641 PMC7485574

[9] Desquesnes M Gonzatti M Sazmand A Thévenon S Bossard G Boulangé A. A review on the diagnosis of animal trypanosomoses. Parasit Vectors. (2022) 15:64. doi: 10.1186/s13071-022-05190-1, PMID: 35183235 PMC8858479

[10] Hassan-Kadle AA Ibrahim AM Nyingilili HS Yusuf AA Vieira TS Vieira RF. Parasitological, serological and molecular survey of camel trypanosomiasis in Somalia. Parasit Vectors. (2019) 12:1–6. doi: 10.1186/s13071-019-3853-5 31864389 PMC6925896

[11] Desquesnes M Sazmand A Gonzatti M Boulangé A Bossard G Thévenon S. Diagnosis of animal trypanosomoses: proper use of current tools and future prospects. Parasit Vectors. (2022) 15:235. doi: 10.1186/s13071-022-05352-1, PMID: 35761373 PMC9238167

[12] Kangethe RT Winger EM Settypalli TBK Datta S Wijewardana V Lamien CE. Low dose gamma irradiation of *Trypanosoma evansi* parasites identifies molecular changes that occur to repair radiation damage and gene transcripts that may be involved in establishing disease in mice post-irradiation. Front Immunol. (2022) 13:852091. doi: 10.3389/fimmu.2022.852091, PMID: 35634275 PMC9136415

[13] Nantulya VM. Trypanosomiasis in domestic animals: the problems of diagnosis. Rev Sci Tech. (1990) 9:357–67. doi: 10.20506/rst.9.2.507, PMID: 2132685

[14] Ngaira JM Bett B Karanja SM Njagi ENM. Evaluation of antigen and antibody rapid detection tests for Trypanosoma evansi infection in camels in Kenya. Vet Parasitol. (2003) 114:131–41. doi: 10.1016/S0304-4017(03)00112-2, PMID: 12781475

[15] Kim J Álvarez-Rodríguez A Li Z Radwanska M Magez S. Recent progress in the detection of Surra, a neglected disease caused by *Trypanosoma evansi* with a one health impact in large parts of the tropic and sub-tropic world. Microorganisms. (2023) 12:44. doi: 10.3390/microorganisms12010044, PMID: 38257871 PMC10819111

[16] Benfodil K Büscher P Abdelli A Van Reet N Mohamed-Herif A Ansel S. Comparison of serological and molecular tests for detection of Trypanosoma evansi in domestic animals from Ghardaïa district, South Algeria. Vet Parasitol. (2020) 280:109089. doi: 10.1016/j.vetpar.2020.109089, PMID: 32222595

[17] Thrusfield M. Veterinary epidemiology. Hoboken, NJ: John Wiley & Sons (2018).

[18] Benaissa MH Mimoune N Bentria Y Kernif T Boukhelkhal A Youngs CR. Seroprevalence and risk factors for Trypanosoma evansi, the causative agent of Surra, in the dromedary camel (*Camelus dromedarius*) population in southeastern Algeria. Onderstepoort J Vet Res. (2020) 87:e1–9. doi: 10.4102/ojvr.v87i1.1891, PMID: 33354976 PMC7756738

[19] Lemecha H Lidetu D Hussein I. Prevalence and distribution of camel trypanosomosis in the semi-arid and arid Awash Valley of Ethiopia. Ethiop J Anim Prod. (2008) 8:1–9.

[20] Sobhy HM Barghash SM Behour TS Razin EA. Seasonal fluctuation of trypanosomiasis in camels in north-West Egypt and effect of age, sex, location, health status and vector abundance on the prevalence. Beni-Suef Univ J Basic Appl Sci. (2017) 6:64–8. doi: 10.1016/j.bjbas.2017.01.003

[21] Ismail Mohamoud A. Sero-prevalence study of camel trypanosomiasis in selected villages of Galkayo, Somalia. Open J Vet Med. (2017) 7:31–7. doi: 10.4236/ojvm.2017.74004

[22] Tehseen S Jahan N Qamar MF Desquesnes M Shahzad MI Deborggraeve S. Parasitological, serological and molecular survey of *Trypanosoma evansi* infection in dromedary camels from Cholistan Desert, Pakistan. Parasit Vectors. (2015) 8:415. doi: 10.1186/s13071-015-1002-3, PMID: 26259616 PMC4532143

[23] Boushaki D Adel A Dia ML Büscher P Madani H Brihoum BA. Epidemiological investigations on *Trypanosoma evansi* infection in dromedary camels in the south of Algeria. Heliyon. (2019) 5:e02086. doi: 10.1016/j.heliyon.2019.e02086, PMID: 31372547 PMC6656995

