# Supplementary material for: Serological surveillance of Trypanosoma evansi in Kazakhstani camels by complement fixation and formalin gel tests
Source: Front Vet Sci. 2025 Sep 23;12:1661387. doi: 10.3389/fvets.2025.1661387 (PMC12502730; doi:10.3389/fvets.2025.1661387)
Supplement: Supplementary file 1 [file Table_1.DOCX]

**Supplementary Table S1. District-level seroprevalence of T. evansi in camels**

| **Region** | **District** | **CFT** | **FGT** |
| --- | --- | --- | --- |
| Turkestan | Shardara | 2/28 (7.14%; 95% CI: 1.98–22.65) | 4/28 (14.29%; 95% CI: 5.70–31.49) |
| Kyzylorda | Karmakshy | 45/320 (14.06%; 95% CI: 10.68–18.30) | 61/320 (19.06%; 95% CI: 15.14–23.72) |
|  | Kyzylorda | 2/550 (0.36%; 95% CI: 0.10–1.32) | 3/550 (0.55%; 95% CI: 0.19–1.59) |
|  | Syrdarya | 1/265 (0.38%; 95% CI: 0.07–2.11) | 7/265 (2.64%; 95% CI: 1.29–5.35) |
|  | Kazaly | 28/840 (3.33%; 95% CI: 2.32–4.78) | 46/840 (5.48%; 95% CI: 4.13–7.23) |
|  | Aral | 30/670 (4.48%; 95% CI: 3.16–6.32) | 90/670 (13.43%; 95% CI: 11.06–16.24) |
| Mangystau | Munaily | 3/30 (10.00%; 95% CI: 3.46–25.62) | 27/30 (90.00%; 95% CI: 74.38–96.54) |
|  | Mangystau | 1/40 (2.50%; 95% CI: 0.44–13.16) | 29/40 (72.50%; 95% CI: 57.19–83.89) |
|  | Tupkaragan | 1/30 (3.33%; 95% CI: 0.59–16.67) | 9/30 (30.00%; 95% CI: 16.68–47.86) |
